# Geriatric-focused education for community health workers: a dementia training that effectively provides knowledge and skills to address dementia within the scope of practice of this public health workforce

**DOI:** 10.3389/fpubh.2025.1601388

**Published:** 2025-09-01

**Authors:** Kerstin M. Reinschmidt, Zahra A. Alhay, Keith L. Kleszynski, Lee A. Jennings

**Affiliations:** ^1^Department of Health Promotion Sciences, Hudson College of Public Health, University of Oklahoma Health Sciences Center, Oklahoma City, OK, United States; ^2^Department of Geriatric Medicine, College of Medicine, University of Oklahoma Health Sciences Center, Oklahoma City, OK, United States

**Keywords:** community health worker, geriatric, dementia, training, workforce development

## Abstract

**Background:**

In light of the public health crisis of dementia, community health workers (CHWs) have increasingly received national attention as a public health workforce that can support individuals, families, and communities in preventing, recognizing, and living with dementia. With training, CHWs have high potential for making a positive impact on healthy aging and living with dementia. Recognizing the need for a CHW-specific training, the Oklahoma Dementia Care Network (OkDCN) developed the Dementia Training for Community Health Workers. This paper examines the training program’s pedagogical framework and comprehensive evaluation processes, tools, and outcomes.

**Methods:**

This training was developed as a train-the-trainer model combining didactic instruction with adult learning strategies. The training’s content resulted from combining knowledge about CHWs in Oklahoma, National C3 Council recommendations on the CHW scope of work, and evidence-based dementia care strategies. The evaluation design measures effectiveness focused on the first three levels of the Kirkpatrick Model, i.e., CHW training feedback, knowledge and skill acquisition, and application of the training knowledge and resources. The OkDCN collaborated with CHWs to develop and implement the training.

**Results:**

Between June 2020 and March 2024, we conducted 15 trainings with a total of 307 trainees. Data collected on pre/post knowledge, self-efficacy, and post-training feedback were analyzed as a subsample of the total number of trainees. Among this subsample (50%; *n* = 154) both knowledge and some of the self-efficacy items showed statistically significant improvements. Overall, trainees were satisfied with the training in terms of content, format, and delivery. A follow-up survey showed that trainees appreciated the practical value of the training for their jobs and used the information in their day-to-day work, but did not yet conduct dementia trainings in their communities.

**Conclusion:**

As CHWs are tasked with alleviating the public health crisis of dementia, they necessitate geriatric-focused education that is evidence based, workforce-appropriate, and adaptable to diverse communities. Designed specifically for CHWs as a peer-implemented train-the-trainer model, this innovative training contributes to both workforce development and health equity. CHWs who apply or pass on their knowledge can make positive impacts on their communities’ healthy aging.

## Introduction

In light of the public health crisis of dementia, community health workers (CHWs) have increasingly received national attention as a public health workforce that can support individuals, families, and communities in preventing, recognizing, and living with dementia (1–3). Research is emerging to assess the roles and impacts of CHWs in addressing dementia (4, 5). While some call for additional training for this workforce (6), others have already developed dementia trainings for CHWs with a focus on healthy aging and addressing dementia (7–9). Our dementia training for CHWs was developed in Oklahoma (10) and is unique because it builds on the broad CHW scope of work (11, 12) and is designed for peer-trainers within the train-the-trainer model.

Dementia is an umbrella term for chronic, progressive, and irreversible conditions and diseases that share a decline in memory, thinking, and problem-solving all of which impact everyday life activities, behavior, and relationships. Alzheimer’s disease, the most common type of dementia, is the 7th leading cause of death among adults in the U.S., and the 6th leading cause of death among adults 65 years and older (13). The number of Americans living with Alzheimer’s disease is projected to double from an estimated 6.9 million Americans in 2020 to nearly 14 million in 2060 (13). Since the risk for Alzheimer’s dementia increases with age and typically affects those aged 60 or older (13), an aging U.S. population contributes to this upward trend. It is concerning that the largest increases in dementia are expected to burden people from minoritized groups and women (14). Risk factors for these disparities include high prevalence of other chronic conditions, social determinants of health, and women’s higher life expectancy (14).

Dementia creates a human and monetary burden not only for the individuals affected by a decline in cognitive abilities and their caregivers, but also for healthcare systems. In 2020, the number of people in Oklahoma aged 65 and older was estimated at 750,000, comprising 10.8% of the state’s population (15). In 2017, the number of Oklahomans in hospice with a primary diagnosis of dementia was 4,102, or 18% of hospice residents (16). According to the Alzheimer’s Association, 108,000 caregivers in Oklahoma provided a total value of over $3B unpaid dementia care in 2024 (16). Medical costs in 2020 included $516 M in Medicaid for caring for people with Alzheimer’s (16). In 2021, a total of 1,580 Oklahomans died from Alzheimer’s disease, which was a 147.6% Alzheimer’s mortality increase between 2000 and 2021 (16). In 2024, Oklahoma ranked 46th in senior health and 48th out of 50 states for early death among older adults (ages 65–74) (17).

Oklahoma is largely rural, with 72 of 77 counties designated as entirely or partially rural (18) and medically underserved with every county identified as having Health Professional Shortage Areas (HPSA score >17) (19). Only 35% of older adults live within Oklahoma’s two urban areas, leaving 65% geographically distant from specialized geriatric care. In 2024, Oklahoma ranked 45th for geriatrician shortfall, and 43rd in home health care workers (17). In 2021, the state had 26 geriatricians and needed a 557.7% increase to meet the demand in 2050 (16). Oklahoma is classified as a “dementia neurology desert,” i.e., as one of the states with the greatest gap between available neurologists and the number of persons with Alzheimer’s disease and related dementias (20).

With training, CHWs are a public health workforce with high potential for making a positive impact on healthy aging and living with dementia. The term “community health workers” refers to a workforce that is known by many names, including community health representatives (CHRs) who are CHWs working with American Indian communities. Paraphrasing the American Public Health Association’s definition (21), CHWs are frontline public health workers who are trusted members of the communities they serve which allows them to act as links between health/social services and communities. Importantly, CHWs build individual and community capacity by providing health knowledge and self-sufficiency through a variety of roles. CHWs are known to be effective in improving chronic disease management and chronic disease disparities (22–26). Recent evidence also shows that CHWs can play multiple roles that help prevent and manage dementia (5).

Recognizing the need for a dementia training program specifically developed for CHWs, the Oklahoma Dementia Care Network (OkDCN), a Geriatrics Workforce Enhancement Program first funded by the Health Resources and Services Administration in 2019, included CHWs in their aim to address the unmet dementia needs of diverse populations by providing health and public health workforce education. In a previous publication (10), we described the collaborative development and implementation of our dementia training, and the value we placed on CHW leadership in workforce development by designing and implementing a train-the-trainer model. In this paper, we will discuss the training program’s pedagogical framework and comprehensive evaluation processes, tools, and outcomes. The *Dementia Training for Community Health Workers* has been implemented in Oklahoma and beyond for 4 years to provide CHWs with the needed competencies to address dementia within the broad scope of CHW core roles (11, 12).

## Pedagogial framework(s), principles, and standards

OkDCN’s *Dementia Training for Community Health Workers* provides an educational model that aligns with and fills a gap in the broad scope of work for CHWs (11, 12). It not only provides a program for the CHW core skill “Knowledge Base” and its sub-skill “knowledge about pertinent health issues,” but it also uses the 10 CHW core roles as a framework to organize information on how CHWs can address dementia based on each role. Our training is unique because it is a train-the-trainer model that was developed (2019–2020), piloted (2020), and implemented (2020–current) using a collaborative framework (10). The content of the training resulted from combining knowledge about CHWs in Oklahoma, National C3 Council recommendations on the CHW scope of work, and evidence-based dementia care strategies (10). The program includes an introduction to the training, basic information on dementia, CHW roles in addressing dementia, and appendices with training materials and resources. The training combines a train-the-trainer model with didactic instruction and adult learning theory.

Train-the-trainer programs can be effective in disseminating knowledge and information to health and social service professionals (27, 28). In general, organizations with content-area experts develop training materials, tools, and guidelines to train future trainers to provide these trainings within their communities or to professional peers (27, 29). While fidelity of training implementation may be a concern for train-the-trainer models (27), their benefits may disperse this concern. Disseminating knowledge to community-based trainers instead of safeguarding it can sustain knowledge in the community. Train-the-trainer models utilize community social capital, i.e., established social relationships of trust, which can enhance learning and contribute to community empowerment (27, 29).

Our training combines brief lectures with various interactive strategies. Learner engagement is built into the training through videos, case studies, reflections, tools, exercises, team work, and open discussions. Anchored in andragogy, i.e., the theory and practice of teaching adults (30), interactive strategies are specific to the CHW core roles and aim to solicit examples, stories, and discussions of professional expertise and shared lived experience. Thus, by actively engaging in the training, CHWs contribute to their own and their peers’ learning and increase the value of the teaching materials for future application. According to constructivist and transformative learning theories (31), CHWs who construct new knowledge by engaging with peers, integrate this knowledge and thus transforming previously held propositions.

To maintain high standards, we evaluated the effectiveness of the *CHW Dementia Training*. Our evaluation used the first three levels of the Kirkpatrick Model, i.e., CHW feedback to the training (Reaction), knowledge and skill acquisition (Learning), and application of the training knowledge and resources (Behavior) (32).

## Learning environment and pedagogical format

We previously (10) described the collaborative nature of the training development and implementation, and the train-the-trainer model whereby CHWs/CHRs who had taken the training became trainers for their peers in subsequent trainings.

### Setting

The training program was originally developed to be conducted in-person. Due to COVID-19, we moved to a virtual platform. This setting offered flexibility and accessibility, as participants joined from across Oklahoma and other states. Over 4 years, only three trainings took place in-person. Trainings can be delivered virtually, in-person, or as a hybrid with options that include reviewing the recorded information on dementia basics asynchronously before attending the synchronous portion of the training. This training can be delivered in English or Spanish.

### Trainees

The program was developed specifically for CHWs/CHRs with the goal of enhancing their knowledge and skills within the context of dementia care. During trainings, participants routinely shared about their diverse backgrounds and experiences, contributing to a rich learning environment. Recruitment of trainees occurred through the OkDCN website and by directly reaching out to organizations.

### Training team

The training was facilitated by CHWs/CHRs, University of Oklahoma Health Sciences faculty and graduate research assistants (GRAs), and guest speakers. CHWs/CHRs provided the bulk of the training content and led trainees in active participation, while faculty and GRAs supported training logistics. Guest speakers focused on specific resources related to dementia and healthy aging.

### Pedagogical format

The collaborative and interactive training sessions included brief virtual or in-person didactics, group discussions, case studies, reflections, and other activities. The use of different resources, such as videos, enhanced the participants’ learning experience. The learning objectives were designed to equip training participants with the ability to promote brain health, recognize dementia, refer to care and community resources, and support the care of persons with dementia and their family caregivers. While the training section on basic information on dementia took a more didactic approach to instruction, interspersing three brief videos broke up the stream of information and invited participants to reflect on the content and share lived experiences. A recall activity of the 10 early signs and symptoms of Alzheimer’s disease also provided an opportunity for engagement. However, trainees who chose the 1 h video recording of this section did not benefit from these interactions. A pre- and post-test assessment evaluated participants’ knowledge acquisition and self-efficacy before and after engaging with this section on basic dementia knowledge. The longest section of the training introduced and discussed CHW roles in addressing dementia. Each of the 10 CHW core roles was discussed by first introducing the role and its sub-roles (11, 12) before providing specific examples on how to utilize the role to address dementia. Throughout these segments, training participants were engaged through reflections, case studies, exercises, and activities.

## Processes, tools, and results

### Processes and tools

Our training has been accompanied by a comprehensive evaluation plan. Data collected included demographics, pre/post-test knowledge and self-efficacy, post-training feedback, and application of knowledge and resources gained from the training.

#### Demographic characteristics

Demographic information of training participants including gender, age, race, and ethnicity were collected. Over 4 years, the source of the demographic data changed from the Post-Training Feedback survey—available for analysis either as raw data or tables in evaluation summary reports (2020–2022)—to the Pretest Survey (2022–2024) (Table 1). Descriptive statistics, including frequencies and percentages, were used to summarize demographic characteristics.

**Table 1:** Train-the-trainer (TtT) and basic *Dementia Training for Community Health Workers* (June 2020–May 2024).^a^

Funding year, type of training, and month/year of training	Year 1 (2020–2021)					Year 2 (2021–2022)				Year 3 (2022–2023)			Year 4 (2023–2024)			
	TtT Pilot	TtT	Basic 1	Basic 2	Basic 3	TtT	Basic 1	Basic 2	Basic 3	TtT	Basic 1	Basic 2	TtT	Basic 1	Basic 2	Basic 3
	Jun. ‘20	May ‘21	Oct. ‘20	Feb. ‘21	Mar. ‘21	Apr. ‘22	Nov. ‘21	Feb. ‘22	Mar. ‘22	May ‘23	Sep. ‘22	Jan/Feb ‘23	Feb. ‘24	Nov. ‘23	Mar. ‘24	May “24
Training format	Online	Online	Online	Online	Online	Online	Online	Online	Online	In-person	Online	Online	In-person	Online	Online	In-person
Number of participants who completed the training	13	16	16	11	2	6	9	14	9	4	13	96	8	28	19	5
Demographic information source	NA	27 Demo. table	16 Demo. table	17 Demo. table	4 Demo. table	4 Feedback survey	9 Demo. table	19 Feedback survey	16 Feedback survey	4 Pretest survey	12 Feedback survey	97 Pretest survey	7 Pretest survey	46 Pretest survey	29 Pretest survey	8 Pretest survey
Pretest data	9	9	12	10	2	6	NA	25	7	4	14	97	7	46	29	8
Posttest data	9	9	12	10	2	5	NA	24	9	3	13	84	8	31	23	6
Matched pre/post test data	9	9	12	10	2	5	NA	24	7	3	13	72	7	31	23	6
Training feedback	Available in the evaluation summary	Available in the evaluation summary	Available in the evaluation summary	Available in the evaluation summary	Available in the evaluation summary	4	Available in the evaluation summary	19	16	4	12	77	7	31	22	4
^a^Color coding:	Qual data source					Quan data source				Feedback data source			NA, not available			

#### Pre/post-test assessment

The pre/post-tests served to evaluate training participants’ knowledge acquisition and self-efficacy. Dementia training outcomes were measured by a validated 27-item assessment (10, 33). To test for effectiveness, an aggregate analysis was performed of all knowledge, pre- and post-test assessments collected from seven trainings that were conducted over 3 years between April 2022 and March 2024 (Table 1). A paired *t*-test was used to assess the average difference in knowledge from pre-test to post-test measurement and to evaluate the significance of change in the mean knowledge score. Specifically, the difference in knowledge scores was calculated by subtracting pre-test from post-test scores for each question. The null hypothesis was the difference between the paired population means is equal to zero. The alternative hypothesis was the difference between the paired population means is not zero. The self-efficacy instrument was measured by a 10-item modified version (34), however, only the five items most relevant to the training were included in our analysis. The Likert-Type self-efficacy items (1: I can explain the relationship between Alzheimer’s disease and dementia; 2: I can discuss the warning signs of dementia; 3: I know how to get dementia-related services that will help me support my client; 4. Dementia is a natural part of aging, and I cannot do anything about it; 5. I would like to learn more about dementia and aging) were coded into a 3-item scale (1: not at all; 2: somewhat; 3: very much). To test for effectiveness, we used the Wilcoxon Signed-Ranks Test to determine the significance of change in the median self-efficacy for each item individually. The null hypothesis was that the median self-efficacy score of the training participants would not change from pretest to posttest. The alternative hypothesis was that the median posttest self-efficacy of the training participants would be lower at pretest. The level of significance was determined at (*α* = 0.05). Data were analyzed using SAS software version 9.4.

#### Post-training feedback

A mixed methods follow-up assessment measured satisfaction with the training. The analysis was based on nine trainings over 3 years (2022–2024) (Table 1). The survey inquired about satisfaction with training content, format, and training delivery. Survey items were coded on a 4-item scale (1: strongly disagree; 2: disagree; 3: agree; 4: strongly agree). Open-ended post-training feedback questions were used to assess participants’ satisfaction with the dementia training. The questions included feedback on what was deemed the most important information provided in the training, what participants liked most about the training, how participants would improve the training, gauging the training’s effectiveness in terms of increasing CHW/CHR capacity to improve population health, and an opportunity to share any additional feedback. We compiled the participants’ open-ended feedback and analyzed the qualitative data thematically, using NVivo software (35). The lead authors (Kerstin M. Reinschmidt, Zahra A. Alhay, Keith L. Kleszynski) developed deductive and inductive codes independently, subsequently engaged in consensus building to refine and finalize the codes and developed a codebook. All analysts independently coded all qualitative data, then compared their coding to resolve any disagreement through consensus building and ultimately reached inter-coder reliability upwards of 80%. Using the revised coded data, one analyst (Kerstin M. Reinschmidt) identified emerging patterns and themes, which were discussed, revised, and confirmed by the lead authors (36).

#### Training application survey

In 2023 (June 7th–July 14th), 166 *CHW Dementia Training* participants received an email invitation for a brief survey to help assess the benefits and application of the knowledge and resources gained from the training. The anonymous survey covered six key areas, including demographic and workforce characteristics, knowledge related to the dementia training for CHWs/CHRs, satisfaction with the training, use of the training, application of the training, and recommendations. Descriptive statistics, including frequencies and percentages, were used to summarize the six key areas. All analyses were done using SAS software version 9.4. Qualitative data were analyzed to contextualize survey findings from satisfaction responses. Thematic qualitative analysis generated patterns and themes (36).

## Results

Between June 2020 and March 2024, we conducted 15 trainings with a total of 307 trainees who provided demographic data. Data collected on pre/post knowledge, self-efficacy, and training satisfaction were analyzed as a subsample of the total number of training participants. This subsample (50%; *n* = 154) included participants whose demographic data could be matched to the pre- and post-test data (Tables 1, 2).

**Table 2:** Demographics of participants in the *Dementia Training for Community Health Workers* (June 2020–May 2024).

Demographics	CHWs trained (2020–2024), *n* = 307		Pre- and post-test participants, *n* = 154	
Category	Frequency	Percentage	Frequency	Percentage
Gender				
Male	27	9%	14	9%
Female	246	80%	138	90%
Other	2	1%	1	1%
Unknown	31	10%	0	0%
Frequency missing	1	0%	1	1%
Age				
20–29 years	34	11%	18	12%
30–39 years	56	18%	34	22%
40–49 years	55	18%	29	19%
50–59 years	66	21%	36	23%
60 years and older	63	20%	35	23%
Unknown	32	10%	0	0%
Frequency missing	1	0%	2	1%
Race				
White or Caucasian	72	23%	24	16%
White or Caucasian; American Indian or Alaska Native	10	3%	7	4%
White or Caucasian; Black or African American	1	0%	1	1%
White or Caucasian; Native Hawaiian or other Pacific Islander	2	1.0%	2	1%
Black or African American	20	7%	7	5%
Asian	2	1%	1	1%
American Indian or Alaska Native	158	51%	100	65%
Native Hawaiian or other Pacific Islander	21	7%	1	1%
Unknown	20	7%	8	5%
Frequency missing	1	0%	2	1%
Ethnicity				
Hispanic	42	14%	19	12%
Non-Hispanic	210	68%	123	80%
Unknown	43	14%	9	6%
Frequency missing	12	4%	3	2%
Total number of participants	307	100%	154	100%

### Demographics

The majority of training participants in the subsample were female (90%; *n* = 138), American Indian or Alaska Native (65%; *n* = 100), White or Caucasian (16%; *n* = 24), and non-Hispanic (80%; *n* = 123), with fairly balanced age distributions. There were no large differences between the overall training population and the evaluation subsample (Table 2). The training had a wide reach with the majority of participants (54%; *n* = 83) from a range of states across the U.S. and 42% (*n* = 65) from Oklahoma. Most Oklahoma participants represented organizations from rural areas (62%; *n* = 40), while participants from other states more often worked in urban areas (52%; *n* = 43). The majority of participants from Oklahoma as well as other states reported organizational affiliations with American Indian tribal entities (72%; *n* = 111) and health departments (14%; *n* = 21). Among the subsample, all but three (2%) participants provided information about their professional roles. The majority were CHRs (38%; *n* = 58) or CHWs (37%; *n* = 56), CHW/CHR Supervisors (9%; *n* = 13), and 16% (*n* = 24) had other roles (Table 3).

**Table 3:** Reach and employing organizations of participants in the *Dementia Training for Community Health Workers* with matched demographic and pre/post-test data (June 2020–May 2024), *n* = 154.

Reach and number (%) of employing organizationsof participants	State			Total (*n* = 154)
	Oklahoma *n* = 65 (42%)	Other states* *n* = 83 (54%)	Missing *n* = 6 (4%)	
Reach by rural/urban location				
Rural	40 (62%)	36 (43%)	0	76 (49%)
Urban	23 (35%)	43 (52%)	0	66 (43%)
Missing	2 (3%)	4 (5%)	6	12 (8%)
Employment by types of organization				
Tribal entities	48 (74%)	61 (73%)	2 (33%)	111 (72%)
Health departments	10 (15%)	11 (13%)	0 (0%)	21 (14%)
Non-profit organizations	3 (5%)	3 (4%)	0 (0%)	6 (4%)
Hospital or clinic	1 (1%)	5 (6%)	0 (0%)	6 (4%)
Universities	0 (0%)	1 (1%)	0 (0%)	1 (1%)
Missing	3 (5%)	2 (2%)	4 (67%)	9 (6%)

^*^Other states (20): AK, AZ, CA, CO, IL, KS, LA, MD, MI, MN, MT, NE, NV, NM, NY, NC, OR, VA, WI, and WY.

### Changes in knowledge and self-efficacy

Mean dementia knowledge score improved by two points (SD ± 3, *p* = <0.0001) or by 8% (95% CI 5.39–10.68) after completion of the training. Participants also reported greater self-efficacy following the training, specifically greater confidence in their ability to explain the relationship between Alzheimer’s disease and dementia (Item 1, *p* < 0.0001), discuss the warning signs of dementia (Item 2, *p* < 0.0001), and get dementia-related services to support their clients (Item 3, *p* < 0.0001). There was no difference in pre- and post-assessments for the other items; however, at baseline participants already strongly disagreed that dementia was a natural part of aging that they could not impact (Item 4) and strongly agreed that they wanted to learn more about dementia and aging (Item 5) (Table 4).

**Table 4:** Knowledge and efficacy change of participants in the *Dementia Training for Community Health Workers* with matched demographic and pre/post-test data (n = 154) (April 2022 – May 2024).

Differences of knowledge and self-efficacy scores at pretest and posttest (*N* = 154)									
Knowledge^a^	Pretest		Posttest						
	Mean	SD	Mean	SD	D*	SD	% Change	95% CI**	***P***
	23	±4	25	±3	2	±3	8%	5.39 – 10.68	<0.0001
Self-efficacy item^b^	Median		Median		D***				p****
Item 1: I can explain the relationship between Alzheimer's disease and dementia	2		2.5		−1				**<.0001**
Item 2: I can discuss the warning signs of dementia	2		3		−1				**<.0001**
Item 3: I know how to get dementia-related services that will help me support my client	2		2.5		−1				**<.0001**
Item 4: Dementia is a natural part of aging, and I can't do anything about it	1		1		0				0.072
Item 5: I would like to learn more about dementia and aging	3		3		0				0.067

^a^Dementia knowledge test: scores range from 0 to 27, with higher scores indicating greater knowledge about dementia. D*: Mean difference. 95% CI**: Confidence interval of percentage change in dementia knowledge.

^b^Self-efficacy scale: self-efficacy items were rated on a 3-item scale (1: not at all; 2: somewhat; 3: very much). D***: Median difference. p****: Bolded *p*-values indicated significant self-efficacy items.

### Post-training feedback

**Figure 1:**
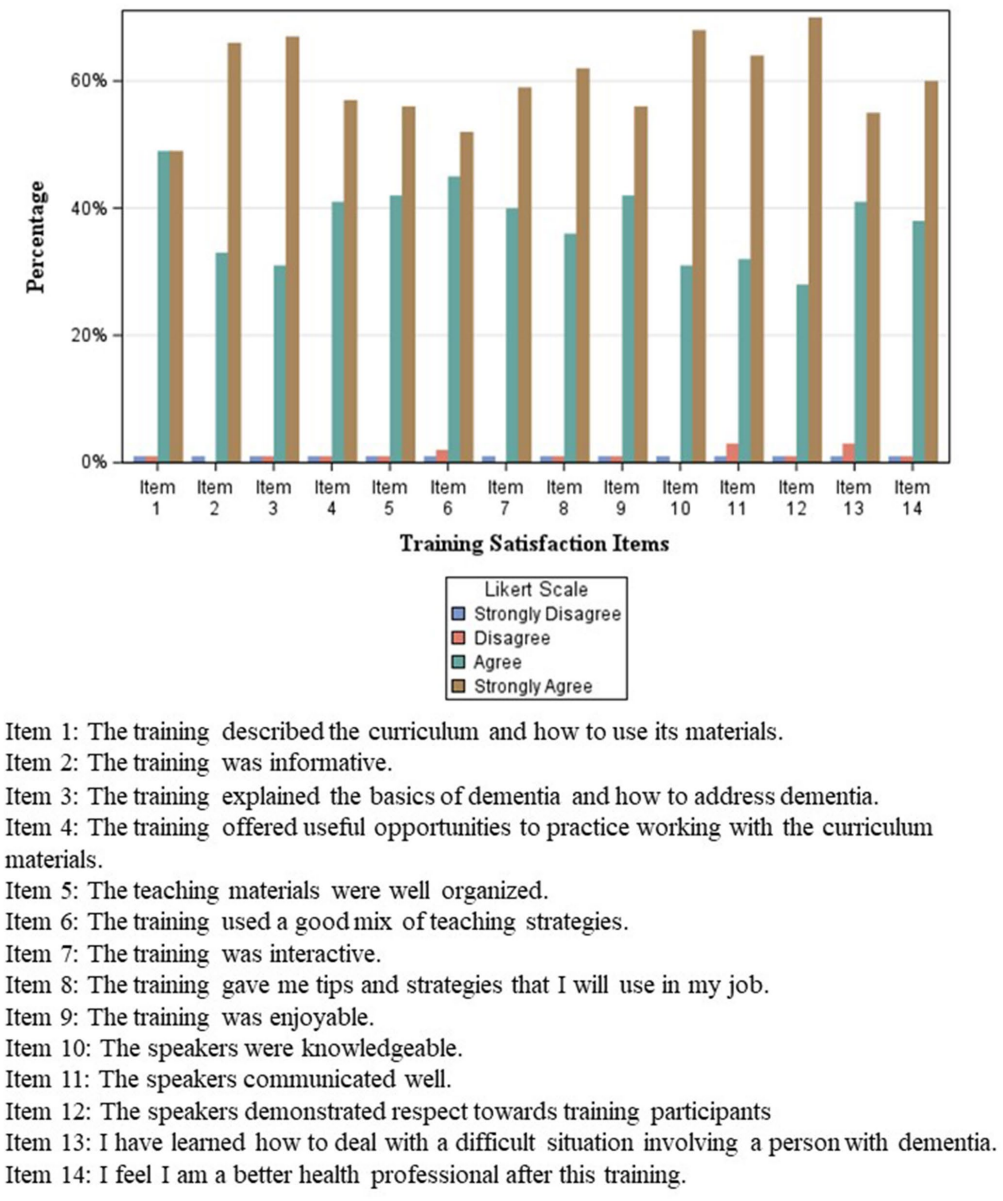
Post-training feedback: satisfaction of participants in the dementia training for CHWs in Oklahoma (*n* = 192) (February 2022–March 2024).

Out of the 307 participants, 192 participants provided post-training feedback between 2022 and 2024. The responses indicated that, overall, trainees were satisfied with the training content, format, and delivery (Figure 1). Approximately two-thirds of participants “strongly agreed” that the training content “explained the basics of dementia and how to address dementia” (67%; *n* = 129), “was informative” (66%; *n* = 127), and “gave them tips and strategies that they will use in their job” (62%; *n* = 119). Over half of participants “strongly agreed” that “the training offered useful opportunities to practice working with the curriculum materials” (57%; *n* = 109) and “the teaching materials were well organized” (56%; *n* = 107). Participants were similarly satisfied with training delivery with 70% (*n* = 134) strongly agreeing that “speakers demonstrated respect toward participants,” 68% (*n* = 131) strongly agreed that “the speakers were knowledgeable,” and 64% (*n* = 123) strongly agreed “the speakers communicated well.”

Supporting and expanding on the quantitative findings, thematic analysis resulted in three main themes, i.e., *training content*, *training presentation*, and *training appreciation*, as described below.

In terms of *training content*, the majority of the participants valued the basic information provided in the training because it helped them better understand dementia and had practical value. Topics of practical value included information on best practices for interacting with persons living with dementia, available resources for clients with dementia and/or their caregivers, and how to apply the information learned to existing programs within their respective organizations. Participants also welcomed the information provided on CHW/CHR roles and how these are related to serving persons living with dementia and/or their caregivers. The quotes below illustrate these sentiments:

“How to pick up on someone’s signs of dementia, what resources might be out there for them, how to handle different situations with a dementia client.”

“We have a new Alzheimer grant… In the past three months I have been on many webinars, trainings and meetings. This was the most helpful and informative of any I have attended. I will definitely be using what I learned in my work as a CHW.”

“I loved all of the CHW roles and the information that explained a little about each role and reflections. It brought awareness of what we are currently doing, what we may need to do, and what we can do.”

In terms of the *training presentation*, most participants provided positive feedback about the training format. They thought the information was presented well and particularly liked the activities, interaction, peer-to-peer learning, and sharing of experiences, which included the shared experiences of the trainers themselves. The following quotes illustrate what participants liked about the training:

“All information was presented well, and everyone was able to share ideas of what resources they have in their communities.”

“Having the reflection questions made me think of what we can do in our roles as CHWs to assist the community.”

“The scenarios that were played out, videos, also allowing questions from the audience.”

Finally, in terms of *training appreciation*, most participants were satisfied with the training, appreciated it being offered, and thought that it was effective. Participants also identified how they anticipated using what they learned in their jobs:

“I got more information than I thought I would. I would say any CHW should take this, great job.”

“I feel this training was very effective, there was questions that were asked that helped me get a better understanding of Dementia and Alzheimer's.”

“I feel that I can efficiently use the information taught to help individuals in need and point them to where they need to go for better help.”

While participants generally were vague about specific recommendations for improvement, some provided suggestions related to time, such as shortening the training or breaking it up into shorter sessions, better accommodating different time zones, and having more breaks. Others recommended having more interactive time, more reflection questions, and breakout sessions. A few participants suggested holding the training in Spanish. Some participants criticized technical issues during trainings, and others expressed their preference for in-person trainings. As one participant stated: *“I think having it in person may have had more conversation from attendees.”* While these comments were not extensive nor echoed by all participants, such criticisms are helpful in terms of altering the training to maximize effectiveness.

### Training application

Among all invited participants (*n* = 166), a total of 43 individuals (26%) responded to the follow-up survey. The majority of all participants (70%; *n* = 30) reported using the knowledge and resources gained in their day-to-day work. They applied this knowledge to enhance client care (93%; *n* = 28), provide referrals to new resources (90%; *n* = 27), share knowledge with colleagues (90%; *n* = 27), review more dementia-related materials (87%; *n* = 26), start referring clients to the Alzheimer’s Association (50%; *n* = 15), ensure access to health and social services (93%; *n* = 28), and help community members to increase knowledge and be self-sufficient (80%; *n* = 24). The majority of participants (98%; *n* = 42) did not conduct their own dementia trainings in their communities. Regarding future plans, about a third (33%; *n* = 14) of participants intended to conduct dementia trainings in their community while two-thirds (67%; *n* = 29) did not have such plans.

Qualitative data showed that the majority of those who planned to conduct trainings in their communities aimed for group presentations for clients, families, or senior centers. Some participants had the goal of providing health education on basics such as signs of dementia by handing out informational pamphlets or attending support groups for caregivers and patients. Among those who had no plans to conduct the training, most participants reported that they believed conducting a training was outside their designated roles or positions, while others wanted to learn more about the topic before considering training within their communities (“*Would like to shadow or assist with a training before I do my own*”). A few participants mentioned barriers such as not having anyone to train, transportation issues, or other responsibilities that prevented them from conducting the training. Yet others noted that they did not see a need for the training due to existing programs (“*It is already in place through Alzheimer’s Association*.”).

## Discussion

### Lessons learned

The increasing burden of dementia has drawn attention to CHWs as a public health workforce to promote brain health and support people living with dementia, their caregivers, and communities in preventing, recognizing, and managing dementia. In order to succeed with this challenging task, CHWs need to be well prepared with competencies that align with their professional scope of work. For this purpose, we developed, implemented, and evaluated a dementia training specifically designed for CHWs (10). This training builds on the CHWs’ broad scope of work, uses adult-appropriate instruction, and utilizes a train-the-trainer model for peer-trainers. The lessons learned during 15 trainings can be presented in alignment with the Kirkpatrick Model (32): CHW Feedback to the Training (Reaction), Knowledge and Skill Acquisition (Learning), and Application of the Training Knowledge and Resources (Behavior).

### CHW feedback on the training (reaction)

Overall, participants were satisfied with the training’s content, format, delivery, and perceived effectiveness. Trainees appreciated the opportunity to increase their basic knowledge of dementia and gain insights on how to serve people living with dementia and/or their caregivers. The practical value of the training made it relevant to their jobs, which reflects principles of andragogy (30). The format likely contributed to this favorable evaluation as opportunities to interact with trainers and peers were provided throughout the training, thus allowing for discussions, applied exercises, and sharing of experiences. Trainees provided positive feedback on the interactive and conversational nature of the training. Providing creative opportunities for learner engagement was crucial, especially since trainings were provided through a virtual platform. CHWs/CHRs who are invited to share examples from their own professional and lived experiences related to dementia and dementia care can also increase learning through peer involvement (30, 31). The fact that the trainers themselves were CHWs/CHRs was an important factor in building trust and relationships with and respect among training participants. Implementing effective CHW trainings thus entails providing knowledge with practical value, offering engaged and interactive training formats, maintaining flexibility in training delivery, and working with peer trainers.

### Knowledge and skill acquisition (learning)

Our dementia training demonstrated small but significant improvements in knowledge and self-efficacy. Train-the-trainer programs that use interactive training methods as well as accompanying training materials such as handouts have been found to be effective in disseminating knowledge to health and social care professionals (28, 29). Using interactive approaches and accompanying materials may thus have contributed to the increased knowledge among our trainees. While change in new knowledge and skill acquisition might seem small, it is likely that trainees already had personal and/or professional knowledge of dementia based on their relatively high baseline dementia knowledge scores, an effect that has been observed previously (29). Thus, while our training shows potential to effectively prepare CHWs to address dementia as related to their scope of work, future trainings could increase the number of days or hours of training, incorporate more interactive segments including role play, offer teach-back exercises, implement trainings in Spanish or other languages, and/or add additional dementia content to further increase dementia knowledge scores.

### Application of the training knowledge and resources (behavior)

While trainees appreciated the practical value of the training for their jobs and used the information in their day-to-day work, the large majority of those who responded to the follow-up survey neither conducted nor planned to conduct dementia trainings in their communities. Those with plans to provide the training mentioned group presentations in the community or disseminating informational hand-outs on the basics of dementia. Reasons for not planning to conduct the training included that trainings were outside of CHW roles, wanting to learn more about the topic first, or encountering barriers. It is not uncommon, however, that those who train as trainers do not actually apply their knowledge by training others (27). Gaining the buy-in of CHW employers may be one way of increasing the continued implementation and amplification of the training as intended by the train-the-trainer model.

### Practical implications

This training has practical implications for CHW workforce development and reaching diverse populations.

#### CHW workforce development

Our training on dementia falls within the CHW core skill of specialized knowledge. This training is timely because CHWs have already begun to play multiple roles in supporting the prevention and management of dementia (5). Utilizing the trust and positive relationships with their clients, CHWs have helped people adopt healthy lifestyle habits such as physical activity and eating a heart healthy diet, which consequently reduces the risk of memory loss and promotes healthy aging (1, 13). As members of the communities they serve, CHWs are already fulfilling the need for a culturally and linguistically diverse workforce (14) that can support family care givers in dementia care by providing appropriate education, support, and skills development (4, 7). CHWs have helped persons with dementia and their caregivers access dementia care services and resources (37, 38), thus reducing barriers to health and social care, especially for those influenced by social determinants of health (14). CHWs have already supported communities by providing awareness about dementia (7) and by offering screening for early detection (39). While these programs have prepared CHWs to address specific dementia needs, our program is designed to prepare CHWs to address dementia within their broad scope of work. Moreover, employers should explore how to recognize and incentivize participation in additional training for CHWs. Continuing education credits, certificates, or career ladder benefits could be appropriate mechanisms for recognition and to promote career advancement.

#### Reaching diverse populations

This dementia training can be implemented with diverse populations, as the customizing to specific populations happens during the training when trainees engage in discussions and exercises (10). Thus, the continued use of the train-the-trainer model will benefit CHWs and their communities in other states when trainers create safe spaces to share culturally relevant narratives.

## Conclusion

As CHWs are tasked with alleviating the public health crisis of dementia, particularly in areas of the U.S. with neurology and geriatrics workforce shortages, they necessitate geriatric-focused education that is evidence-based, workforce-appropriate, and adaptable to diverse communities. The *Dementia Training for Community Health Workers* has shown to be a training tool with high potential as CHWs/CHRs in Oklahoma and beyond have increased their competencies in addressing dementia within the broad scope of CHW core roles. Designed specifically for CHWs as a peer-trainer and train-the-trainer model, this innovative training contributes to both workforce development and health equity. CHWs who apply or pass on their knowledge can make positive impacts on their communities’ healthy aging. Future work will continue to adapt and enhance this training and spread it to new audiences to increase the impact of CHWs on improving dementia care and outcomes.

## Data Availability

The datasets presented in this article are not readily available because the data supporting this article cannot be made available by the authors due to ethical considerations of small data sets. However, the authors can make evaluation instruments available without reservation. Requests to access the datasets should be directed to Kerstin-Reinschmidt@ou.edu.
